# Supplementary material for: Keeping People with Dementia or Mild Cognitive Impairment in Employment: A Literature Review on Its Determinants
Source: Int J Environ Res Public Health. 2020 Jan 29;17(3):842. doi: 10.3390/ijerph17030842 (PMC7037722; doi:10.3390/ijerph17030842)
Supplement: Supplementary file 1 [file ijerph-17-00842-s001.pdf]

Supplementary Material. Search strategy (performed on August 20, 2018)

(dementia OR "Mild cognitive impairment" OR mci) AND ("work performance" OR "work ability" OR "performance appraisal" OR employment) AND (LIMIT-TO (PUBYEAR , 2019) OR LIMIT-TO (PUBYEAR , 2018) OR LIMIT-TO (PUBYEAR , 2017) OR LIMIT-TO (PUBYEAR , 2016) OR LIMIT-TO (PUBYEAR , 2015) OR LIMIT-TO (PUBYEAR , 2014) OR LIMIT-TO (PUBYEAR , 2013) OR LIMIT-TO (PUBYEAR , 2012) OR LIMIT-TO (PUBYEAR , 2011) OR LIMIT-TO (PUBYEAR , 2010)) AND (LIMIT-TO (DOCTYPE , "ar") OR LIMIT-TO (DOCTYPE , "ip") OR LIMIT-TO (DOCTYPE , "sh")) AND (LIMIT-TO (LANGUAGE , "English")) AND (LIMIT-TO (SRCTYPE , "j")) AND (EXCLUDE (LANGUAGE , "Spanish") OR EXCLUDE (LANGUAGE , "Portuguese") OR EXCLUDE (LANGUAGE , "Turkish") OR EXCLUDE (LANGUAGE , "French") OR EXCLUDE (LANGUAGE , "German") OR EXCLUDE (LANGUAGE , "Croatian") OR EXCLUDE (LANGUAGE , "Italian") OR EXCLUDE (LANGUAGE , "Polish")) AND (EXCLUDE (SUBJAREA , "BIOC") OR EXCLUDE (SUBJAREA , "ARTS") OR EXCLUDE (SUBJAREA , "AGRI") OR EXCLUDE (SUBJAREA , "ECON") OR EXCLUDE (SUBJAREA , "ENGI") OR EXCLUDE (SUBJAREA , "PHAR") OR EXCLUDE (SUBJAREA , "ENVI") OR EXCLUDE (SUBJAREA , "COMP") OR EXCLUDE (SUBJAREA , "IMMU") OR EXCLUDE (SUBJAREA , "MATH") OR EXCLUDE (SUBJAREA , "CHEM") OR EXCLUDE (SUBJAREA , "DECI") OR EXCLUDE (SUBJAREA , "CENG") OR EXCLUDE (SUBJAREA , "DENT") OR EXCLUDE (SUBJAREA , "MATE") OR EXCLUDE (SUBJAREA , "ENER") OR EXCLUDE (SUBJAREA , "PHYS") OR EXCLUDE (SUBJAREA , "VETE")) AND (EXCLUDE (EXACTKEYWORD , "Physiology") OR EXCLUDE (EXACTKEYWORD , "Schizophrenia") OR EXCLUDE (EXACTKEYWORD , "Multiple Sclerosis") OR EXCLUDE (EXACTKEYWORD , "Home Care") OR EXCLUDE (EXACTKEYWORD , "Pathophysiology") OR EXCLUDE (EXACTKEYWORD , "Nursing Home") OR EXCLUDE (EXACTKEYWORD , "Child") OR EXCLUDE (EXACTKEYWORD , "Traumatic Brain Injury") OR EXCLUDE (EXACTKEYWORD , "Human Experiment") OR EXCLUDE (EXACTKEYWORD , "Long Term Care") OR EXCLUDE (EXACTKEYWORD , "Human Immunodeficiency Virus Infection") OR EXCLUDE (EXACTKEYWORD , "Cerebrovascular Accident") OR EXCLUDE (EXACTKEYWORD , "Mortality") OR EXCLUDE (EXACTKEYWORD , "Health Personnel Attitude") OR EXCLUDE (EXACTKEYWORD , "Attitude Of Health Personnel") OR EXCLUDE (EXACTKEYWORD , "Interviews As Topic") OR EXCLUDE (EXACTKEYWORD , "Stroke")) AND (EXCLUDE (EXACTKEYWORD , "Ethnology") OR EXCLUDE (EXACTKEYWORD , "Health Care Personnel"))
